# Patient Satisfaction with Process-Related Aspects of Outpatient Physiotherapy Care: Preliminary Insights from Croatia

**DOI:** 10.3390/healthcare14193310

**Published:** 2026-10-04

**Authors:** Maroje Višić, Ivana Kos Tolić, Iva Lončarić Kelečić, Jasna Mesarić

**Affiliations:** 1 Faculty of Health Sciences, Libertas International University, 10000 Zagreb, Croatia; mvisic@libertas.hr; 2 Department of Physical Medicine and Rehabilitation, Zagreb-West Health Center, 10000 Zagreb, Croatia; ivana.tolic@dzz-zapad.hr; 3 Physical Therapy Department, University Clinical Hospital Center Zagreb, 10000 Zagreb, Croatia

**Keywords:** outpatient physiotherapy, patient satisfaction, ambulatory care, communication, quality of health care, primary care, cross-sectional study

## Abstract

**Background:** Patient satisfaction is an important indicator of healthcare quality, yet evidence on satisfaction with outpatient physiotherapy (OPT) in Croatian publicly funded primary care settings remains limited. **Methods:** This exploratory cross-sectional study examined patient evaluations of process-related aspects of OPT care and explored factors associated with variability in both composite and item-level ratings. A pragmatic composite process-related satisfaction score was calculated as the mean of 15 questionnaire items. Descriptive statistics, non-parametric group comparisons, exploratory multiple linear regression, and proportional-odds ordinal logistic regression sensitivity analyses were performed. **Results:** The final sample included 151 participants. Patient evaluations were highly favourable, with a median composite score of 4.87 (IQR = 0.43) and a mean of 4.69 ± 0.44. The highest ratings were observed for professional competence (4.91 ± 0.29), friendliness (4.89 ± 0.34), attentiveness (4.87 ± 0.39), and personalised treatment (4.84 ± 0.37). Greater variability and comparatively lower ratings were observed for self-introduction (4.38 ± 1.11), side-effect information (4.41 ± 1.02), and treatment explanation (4.52 ± 0.86). Educational attainment showed a nominal association with the composite score in bivariate analysis, but this did not remain statistically significant after correction for multiple testing. In exploratory multivariable analyses, male sex was nominally associated with higher self-introduction ratings, while previous treatment at the same facility was nominally associated with lower treatment-explanation ratings; however, no predictor-level association remained statistically significant after Holm correction. Explained variance across the linear regression models was modest (adjusted R^2^ = 0.035–0.085). **Conclusions:** Overall, patients reported highly favourable evaluations of process-related OPT care, while selected communication- and information-related items showed greater variability than the composite score. These preliminary findings support complementing composite scores with item-level assessment when examining patients’ experiences of outpatient physiotherapy care.

## 1. Introduction

Patient satisfaction is increasingly recognized as an important indicator of healthcare quality, particularly in rehabilitation settings, where treatment often involves repeated interactions, active patient participation, and the development of an ongoing therapeutic relationship [1,2]. In physiotherapy, satisfaction extends beyond symptom improvement alone and reflects patients’ evaluations of communication and information provision, interpersonal interactions, and physiotherapists’ professional competence [3,4].

Compared with many other healthcare encounters, physiotherapy is characterised by prolonged therapist–patient contact and continuous patient engagement in treatment activities, which may make aspects of communication and the therapeutic relationship particularly important determinants of satisfaction [5,6]. Previous research has consistently shown that patients value clear explanations, attentiveness, empathy, trust, respect, and involvement in care decisions, while dissatisfaction is often associated with poor communication and insufficiently individualised care [7,8,9]. Communication ability has repeatedly emerged as one of the most valued characteristics of a “good” physiotherapist from the patient perspective [7].

Patient satisfaction in physiotherapy is shaped by clinical, interpersonal, organisational, and contextual factors [4,10]. Importantly, a high overall satisfaction score may coexist with variability across specific aspects of care, suggesting that global satisfaction measures may not fully reflect differences in how individual aspects of care are experienced by patients. Consequently, patient satisfaction should not be viewed as a single homogeneous construct.

Despite increasing international interest in patient satisfaction in physiotherapy, the literature remains methodologically heterogeneous, including variability in the instruments used to assess satisfaction [11,12].

Studies from different healthcare settings have examined patient satisfaction with outpatient physiotherapy services, including hospital-based services in Ethiopia and Nigeria [13,14]; however, evidence remains context-dependent, and comparable data from Croatian publicly funded outpatient physiotherapy settings are limited. Published Croatian research has examined service quality and patient/customer satisfaction in selected healthcare settings; however, evidence specifically addressing patient satisfaction with publicly funded outpatient physiotherapy appears limited [15]. We identified no previous Croatian study examining both composite and item-level satisfaction patterns in publicly funded outpatient physiotherapy.

Therefore, the present study aimed to explore patient satisfaction with process-related aspects of outpatient physiotherapy care, including communication, information provision, interpersonal interactions, and physiotherapist professional conduct within a publicly funded outpatient physiotherapy (OPT) setting in Croatia. In addition, a pragmatic composite process-related satisfaction score was calculated as a summary indicator of patients’ evaluations across the questionnaire items. The study was guided by the following research questions (RQs):

RQ1: What are the levels and patterns of patient satisfaction across questionnaire items assessing process-related aspects of outpatient physiotherapy care in a publicly funded physiotherapy setting?

RQ2: How does the pragmatic composite process-related satisfaction score differ according to sociodemographic and treatment-related characteristics, and which factors are independently associated with variability in the composite score and in the individual satisfaction items showing the greatest response variability?

## 2. Materials and Methods

### 2.1. Study Design

This cross-sectional, single-centre study was conducted between April and May 2025 at the Department of Physical Medicine and Rehabilitation, Zagreb-West Health Centre, Croatia. The study was designed as an exploratory investigation of patient satisfaction within routine OPT care. The study was conducted in accordance with the Declaration of Helsinki [16] and received approval from the institutional Ethics Committee prior to data collection. The reporting of this cross-sectional study was guided by the Strengthening the Reporting of Observational Studies in Epidemiology (STROBE) guidelines [17].

### 2.2. Participants

As this was an exploratory cross-sectional study, no formal a priori sample size calculation was performed. Participants were recruited using convenience sampling within routine outpatient physiotherapy practice during the study period. The department employed 10 physiotherapists working across two shifts, all of whom participated in questionnaire distribution. Physiotherapists were not restricted to inviting only patients under their own care; eligible patients treated by other physiotherapists within the department could also be invited to participate. A total of 155 patients were invited to participate and received a questionnaire, and none declined participation. All 155 questionnaires were returned. Four were excluded because they were incomplete, resulting in a final analytic sample of 151 participants (97.4% of distributed questionnaires). The total number of potentially eligible patients attending the department during the recruitment period was not prospectively recorded for study purposes; therefore, the proportion of the overall eligible patient population that was invited to participate could not be determined.

Eligible participants were adults attending outpatient physiotherapy who had received physiotherapy treatment for at least seven consecutive days. Inclusion criteria were: (i) age 18 years or older; (ii) participation in physiotherapy treatment for at least seven consecutive days; (iii) ability to independently read and complete the questionnaire; and (iv) provision of written informed consent. Exclusion criteria were: (i) age younger than 18 years; (ii) physiotherapy treatment duration shorter than seven days; and (iii) inability to provide informed consent or independently complete the questionnaire. Participation was voluntary and anonymous and did not influence ongoing treatment or clinical care.

### 2.3. Measures and Variable Operationalisation

Data were collected using an anonymous paper-based questionnaire developed specifically for the present study. The questionnaire collected sociodemographic and treatment-related information and included 15 items assessing process-related aspects of outpatient physiotherapy care. Questionnaires were completed on-site during the later stages of physiotherapy treatment. After completion, participants placed their questionnaires in a designated collection box for completed questionnaires rather than returning them directly to the physiotherapist who had distributed them. Staff provided procedural assistance where required but did not influence participants’ responses.

Sociodemographic variables included sex, age, educational attainment, and employment status. Age was collected using predefined 10-year categories (18–27, 28–37, 38–47, 48–57, 58–67, 68–77, 78–87, and ≥88 years). For inferential analyses, adjacent categories were subsequently combined into three broader age groups (18–47, 48–67, and 68–87 years) to reduce sparsity and improve model stability. No participants were recorded in the ≥88-year category. Educational attainment was initially recorded in four categories: no formal education, secondary education, higher education, and university education. For inferential analyses, education was dichotomised into lower educational attainment (no formal or secondary education) and higher educational attainment (higher or university education) to reduce sparsity and improve model stability. Treatment-related variables included previous lifetime physiotherapy experience and previous treatment at the same facility, both coded as binary variables.

Self-reported treatment condition was collected as an open-ended response and subsequently categorised into broader clinically meaningful groups to facilitate descriptive presentation and reduce sparsity among low-frequency conditions. Categorisation was performed collaboratively by the research team according to the predominant anatomical region and clinical presentation. The resulting categories were spine disorder, lower extremity disorder, upper extremity disorder, mixed multisite musculoskeletal pain, postoperative/post-traumatic condition, rheumatologic/systemic condition, neurological condition, and other condition.

The study-specific questionnaire comprised 15 items assessing patients’ evaluations of process-related aspects of outpatient physiotherapy care (Supplementary Table S1). In the present study, “process-related aspects” is used as an overarching descriptive term encompassing questionnaire items addressing communication, information provision, interpersonal interaction, and professional conduct during physiotherapy care; these aspects were not prespecified or scored as separate subscales. Questionnaire content was developed through literature review and expert discussion. The conceptual scope of the questionnaire was informed by the physiotherapy patient-satisfaction literature, particularly evidence highlighting communication, information provision, interpersonal interaction, individualised care, and perceived professional competence as relevant aspects of patients’ evaluations of physiotherapy care [3,4,7,18]. Based on this broader conceptual framework and the clinical experience of the research team, concepts considered relevant to the study setting were operationalised into 15 study-specific items. The individual items were not directly derived from, adapted from, or reproduced from these sources or from an existing validated questionnaire. The resulting questionnaire had not undergone prior formal psychometric validation.

The 15 questionnaire items used two five-point response formats according to item wording. Items 1–5 used an evaluative response scale ranging from 1 (“very poor”) to 5 (“excellent”), whereas Items 6–15 used an agreement scale ranging from 1 (“strongly disagree”) to 5 (“strongly agree”). Higher scores consistently represented more favourable evaluations of care, and no items were reverse-coded. The complete wording of all items and their corresponding response formats is provided in Supplementary Table S1.

For descriptive and exploratory analyses, a pragmatic composite process-related satisfaction score was calculated as the arithmetic mean of all 15 item scores. Because the questionnaire used two response formats, had not undergone prior formal psychometric validation, and its dimensional structure was examined only exploratorily in the present sample, the composite score was treated as a pragmatic summary indicator rather than as a validated measure of a single latent construct. No separate subscale scores were calculated.

### 2.4. Statistical Analysis

All analyses were based on complete cases, as no missing questionnaire data were observed. Continuous variables were summarised using means and standard deviations (SDs), medians and interquartile ranges (IQRs), and observed ranges where appropriate, whereas categorical variables were presented as frequencies and percentages. Distributional characteristics of the satisfaction variables were assessed using the Shapiro–Wilk test and visual inspection of their distributions. Given the pronounced ceiling effects, negative skewness, and deviations from normality, non-parametric methods were used for bivariate group comparisons.

Differences in the pragmatic composite process-related satisfaction score according to sex, previous physiotherapy experience, and previous treatment at the same facility were examined using two-sided Mann–Whitney U tests with asymptotic inference, correction for tied ranks, and continuity correction. Differences according to age group and educational attainment were assessed using the Kruskal–Wallis test. Effect sizes were expressed as rank-biserial correlations (r_rb) for Mann–Whitney U tests and rank epsilon-squared (ε^2^) for Kruskal–Wallis tests, with 95% confidence intervals (CIs). Rank-biserial correlation was calculated as r_rb = 1 − 2U/(*n_1_n_2_*), with positive values indicating higher ranks in the second-listed group relative to the first-listed group. Rank epsilon-squared was calculated as ε^2^ = H/(*N* − 1) and represents a non-directional omnibus effect-size measure. To account for multiple testing across the five bivariate comparisons of the composite score, *p*-values were adjusted using the Holm method. Both unadjusted and Holm-adjusted *p*-values are reported.

Because the questionnaire was developed specifically for the present study and had not undergone prior formal psychometric validation, its dimensional structure was explored using exploratory factor analysis (EFA). Given the ordinal nature of the item responses, EFA was based on a polychoric correlation matrix. Sampling adequacy and factorability were evaluated using the Kaiser–Meyer–Olkin (KMO) measure and Bartlett’s test of sphericity. The number of factors to retain was determined using factor-based parallel analysis, and an oblique (oblimin) rotation was applied to allow correlations among factors. The EFA was regarded as exploratory because of the sample size, pronounced ceiling effects, sparse lower response categories, and marginal overall sampling adequacy. Internal consistency was evaluated using raw and standardised Cronbach’s alpha and McDonald’s omega coefficients, with 95% CIs, together with the average inter-item correlation [19,20]. These coefficients were interpreted as indices of internal consistency rather than as evidence of unidimensionality or construct validity.

In addition to the composite score, the three individual items with the largest observed SDs—self-introduction, side-effect information, and treatment explanation—were selected post hoc as exploratory item-level outcomes. Multiple linear regression models were fitted separately for the composite score and each of these three item-level outcomes. Each model included sex, age group, educational attainment, previous physiotherapy experience, and previous treatment at the same facility as predictors. The predictor set was deliberately restricted because of the exploratory nature of the study and to reduce the risk of model overfitting [21]. Employment status and presenting condition were not included in the regression models because several categories contained few participants, and their inclusion would have substantially increased the number of model parameters relative to the available sample size. Regression assumptions and model diagnostics were evaluated using residual and Q–Q plots, influence diagnostics, and collinearity statistics, including tolerance and variance inflation factor (VIF) values [22,23]. Results are presented as unstandardised regression coefficients (B), standard errors (SEs), 95% CIs, *p*-values, and model fit indices, including R^2^, adjusted R^2^, and the F-statistic.

Because the three item-level outcomes were ordinal five-category responses and exhibited substantial ceiling effects, proportional-odds ordinal logistic regression models were additionally fitted as sensitivity analyses using the same predictor set and reference categories as the corresponding linear regression models. Odds ratios (ORs) with 95% CIs were calculated, with ORs greater than 1 indicating higher odds of reporting a response in a more favourable category. The proportional-odds assumption was explored by comparing coefficients across cumulative binary logistic models fitted at each response threshold. Sparse observations in the lower response categories produced unstable threshold-specific estimates and evidence of separation or quasi-separation in some models, precluding a reliable formal assessment of the proportional-odds assumption. Accordingly, the ordinal models were treated as exploratory sensitivity analyses and interpreted cautiously.

Because several regression outcomes showed skewed distributions and ceiling effects, bootstrap analyses were additionally performed to supplement conventional inference for the linear regression coefficient estimates. Bootstrap inference was based on 5000 resamples. Bias-corrected and accelerated (BCa) 95% CIs and bootstrap *p*-values, as implemented in JASP, were obtained for the regression coefficients. To address multiplicity across the linear regression analyses, Holm-adjusted *p*-values were calculated across the 24 predictor tests from the four models (four outcomes × six predictor coefficients), excluding intercepts. For the three ordinal logistic sensitivity models, Holm-adjusted *p*-values were calculated separately across the corresponding 18 predictor tests (three outcomes × six predictor coefficients). Both unadjusted and Holm-adjusted *p*-values are reported, and all regression analyses were interpreted as exploratory.

All statistical tests were two-sided. An unadjusted *p* < 0.05 was considered nominally statistically significant, whereas conclusions accounting for multiplicity were based on Holm-adjusted *p*-values. Analyses were conducted using Jeffreys’ Amazing Statistics Program (JASP, version 0.95.4.0; JASP Team, University of Amsterdam, Amsterdam, The Netherlands) and R within the JASP environment.

## 3. Results

### 3.1. Participant Characteristics

A total of 151 participants were included in the analysis. The sample predominantly consisted of females (68.9%), individuals aged 48–67 years (60.3%), and participants with secondary education (66.9%). Most participants were employed (65.6%). Regarding presenting conditions, the largest subgroup consisted of patients with spine disorders (*n* = 45, 29.8%), followed by mixed multisite musculoskeletal pain (*n* = 28, 18.5%). Post-traumatic and postoperative conditions accounted for 24 participants (15.9%), while lower extremity disorders and upper extremity disorders were reported by 21 (13.9%) and 19 (12.6%) participants, respectively. Rheumatologic/systemic conditions were less frequent (*n* = 9, 6.0%), whereas neurological and other conditions were uncommon within the sample (*n* = 3, 2.0% and *n* = 2, 1.3%, respectively). Previous physiotherapy experience was reported by 28.5% of participants, while 42.4% indicated prior treatment within the same facility. Detailed participant characteristics are presented in Table 1.

### 3.2. Descriptive Statistics of Patient Satisfaction

Patients reported highly favourable evaluations across all questionnaire items assessing process-related aspects of outpatient physiotherapy care (Table 2). Median values were 5.00 for all individual items, indicating a pronounced concentration of responses at the upper end of the response scales. The highest mean ratings were observed for professional competence (4.91 ± 0.29), friendliness (4.89 ± 0.34), attentiveness (4.87 ± 0.39), personalised treatment (4.84 ± 0.37), and respect for privacy (4.83 ± 0.44). In contrast, self-introduction (4.38 ± 1.11), side-effect information (4.41 ± 1.02), and treatment explanation (4.52 ± 0.86) showed comparatively lower mean ratings and the greatest response variability, as reflected by the largest SDs. These three items were therefore selected post hoc as item-level outcomes for exploratory multivariable analyses. The pragmatic composite process-related satisfaction score had a median of 4.87 (IQR = 0.43) and a mean of 4.69 ± 0.44. Item-level response distributions demonstrated pronounced ceiling effects, with the proportion of participants selecting the maximum response category ranging from 64.2% for communication time to 90.7% for professional competence. Complete response distributions across all five response categories are provided in Supplementary Table S2.

### 3.3. Exploratory Factor Analysis and Internal Consistency

Factor-based parallel analysis suggested retention of three factors, with observed eigenvalues exceeding the corresponding simulated eigenvalues for the first three factors and falling below the simulated value for Factor 4. The first unrotated factor accounted for 69.8% of the variance, while the three-factor solution accounted for 80.7% cumulatively. The retained factors were moderately to strongly correlated (r = 0.513–0.711). However, overall sampling adequacy was marginal (KMO = 0.510), and several item-level measures of sampling adequacy were below 0.50. Together with the pronounced ceiling effects and sparse lower response categories, these findings limited the stability and interpretability of the factor solution. Accordingly, the EFA was considered exploratory and was not used to define or score separate subscales.

Internal consistency was high. Raw coefficients were Cronbach’s α = 0.914 (95% CI 0.899–0.930) and McDonald’s ω = 0.927 (95% CI 0.909–0.944), while the corresponding standardised coefficients were α = 0.929 (95% CI 0.913–0.945) and ω = 0.930 (95% CI 0.914–0.946). The average inter-item correlation was 0.467. These findings were interpreted as evidence of internal consistency rather than as evidence of unidimensionality or construct validity. Full EFA and internal consistency results are provided in Supplementary Table S3.

### 3.4. Bivariate Analyses

In the unadjusted bivariate analyses, no statistically significant differences in the pragmatic composite process-related satisfaction score were observed according to sex, age group, previous physiotherapy experience, or previous treatment at the same facility (Table 3). Educational attainment showed a nominal association with the composite score, with participants with lower educational attainment reporting higher scores than those with higher educational attainment (4.73 ± 0.42 vs. 4.59 ± 0.47; H = 5.304, *p* = 0.021, ε^2^ = 0.035, 95% CI 0.002–0.118). However, this association did not remain statistically significant after Holm adjustment (pHolm = 0.105). Thus, none of the five bivariate comparisons remained statistically significant after adjustment for multiple testing.

### 3.5. Exploratory Multivariable Analyses of Process-Related Satisfaction Outcomes

Four exploratory multiple linear regression models were fitted using the same set of sociodemographic and previous-treatment predictors (Table 4). The model for the pragmatic composite process-related satisfaction score explained 7.4% of the variance (adjusted R^2^ = 0.035) and was not statistically significant at the model level, F(6, 144) = 1.915, *p* = 0.082. None of the individual predictors were nominally associated with the composite score.

Model diagnostics indicated no evidence of problematic multicollinearity (tolerance = 0.645–0.961; VIF = 1.041–1.550), and no influential cases were identified based on Cook’s distance. Given the skewed outcome distributions and pronounced ceiling effects, conventional regression estimates were supplemented with BCa bootstrap inference.

The self-introduction model explained 12.2% of the variance (adjusted R^2^ = 0.085) and was statistically significant at the model level, F(6, 144) = 3.333, *p* = 0.004. Male sex showed a nominal positive association with self-introduction ratings (B = 0.488, 95% CI 0.105 to 0.870, *p* = 0.013; BCa 95% CI 0.173 to 0.845); however, this association did not remain statistically significant after correction for multiple testing (pHolm = 0.312).

The side-effect-information model explained 7.8% of the variance (adjusted R^2^ = 0.039) and was not statistically significant at the model level, F(6, 144) = 2.026, *p* = 0.066. No individual predictor reached the nominal significance threshold. The treatment-explanation model explained 9.5% of the variance (adjusted R^2^ = 0.057) and was statistically significant at the model level, F(6, 144) = 2.505, *p* = 0.025. Previous treatment at the same facility showed a nominal inverse association with treatment-explanation ratings (B = −0.432, 95% CI −0.851 to −0.013, *p* = 0.043). However, the corresponding BCa 95% CI included zero (−0.988 to 0.025), the bootstrap *p*-value was 0.070, and the association did not remain statistically significant after Holm correction (pHolm = 0.989). No predictor-level association across the four linear models remained statistically significant after correction for multiple testing.

Because the three item-level outcomes were ordinal five-category responses with pronounced ceiling effects, proportional-odds ordinal logistic regression models were fitted as sensitivity analyses (Table 5). The overall pattern was broadly consistent with the linear regression analyses. Male sex was nominally associated with higher self-introduction ratings (OR = 2.74, 95% CI 1.17 to 6.41, *p* = 0.021), whereas previous treatment at the same facility was nominally associated with lower treatment-explanation ratings (OR = 0.35, 95% CI 0.13 to 0.96, *p* = 0.041). Neither association remained statistically significant after Holm correction across the 18 ordinal predictor tests (pHolm = 0.372 and 0.704, respectively), and no other predictor-level association reached the nominal significance threshold. Threshold-specific cumulative binary models produced unstable estimates for some contrasts because of sparse observations in the lower response categories, including evidence of separation or quasi-separation. Consequently, the proportional-odds assumption could not be assessed reliably, and the ordinal models were interpreted as exploratory sensitivity analyses rather than confirmatory analyses.

## 4. Discussion

The present study explored patient evaluations of process-related aspects of outpatient physiotherapy care in a publicly funded primary care setting in Croatia. Overall, evaluations were highly favourable across questionnaire items. However, despite uniformly high median ratings and a high pragmatic composite process-related satisfaction score, selected items demonstrated greater response variability, particularly physiotherapist self-introduction, provision of side-effect information, and treatment explanation. These findings should not be interpreted as indicators of dissatisfaction, as ratings remained generally favourable across all items. Rather, they indicate that patient evaluations were less uniform for selected communication- and information-related items than for other aspects of care.

This interpretation is consistent with the conceptual work of Ng and Luk, who described patient satisfaction as a multidimensional construct encompassing provider attitude, technical competence, accessibility, and efficacy [3]. It also aligns with the meta-synthesis by Rossettini et al., which identified patient satisfaction in outpatient musculoskeletal physiotherapy as being shaped by clinical outcomes, physiotherapist features, patient characteristics, therapeutic relationship factors, treatment features, and healthcare-setting characteristics [4]. Accordingly, the present findings support the value of examining patient evaluations beyond global or composite summary scores alone.

With respect to the first research question, participants generally evaluated OPT care very positively. The highest ratings were observed for professional competence, friendliness, attentiveness, personalised treatment, and respect for privacy. Similar patterns of generally favourable patient evaluations have been reported in previous physiotherapy studies. Casserley-Feeney et al. found high satisfaction among patients receiving private physiotherapy for musculoskeletal pain [11], while Durmaz et al. reported that a large proportion of patients treated in outpatient musculoskeletal physiotherapy settings were satisfied or very satisfied with care [24]. High levels of patient satisfaction have also been reported in advanced practice physiotherapy models for musculoskeletal conditions, with a systematic review by Desmeules et al. finding satisfaction with physiotherapist-led advanced practice care to be generally comparable to or higher than that reported for usual physician-led care [25].

The favourable evaluations observed in the present study may partly reflect the relational and interactive nature of physiotherapy care. Physiotherapy differs from many other healthcare encounters because it often involves repeated therapist–patient interactions, active patient participation, physical contact, and longer treatment sessions [2,6,12]. These characteristics may increase the relevance of interpersonal and communication-related aspects of care to patients’ overall evaluations.

The present findings are also consistent with literature highlighting communication, empathy, explanation, and patient involvement as important components of physiotherapy care. Potter et al. identified communication ability as one of the most important characteristics of a “good” physiotherapist, while positive physiotherapy evaluations were frequently associated with effective communication and involvement in management [7]. Dhar and Porecha similarly highlighted empathy, active listening, and clear communication as contributors to patient trust, engagement, and satisfaction [9]. Stiller and Wiles reported that friendliness, empathy, explanation of assessment and treatment, respect for privacy, and adaptation of treatment to individual needs were perceived as important aspects of physiotherapy care [18]. The high ratings for attentiveness, friendliness, professional competence, personalised treatment, and respect for privacy in the present study are broadly consistent with these observations.

At the same time, the items showing the greatest variability primarily concerned communication and information provision. Self-introduction, side-effect information, and treatment explanation had comparatively lower mean ratings and larger standard deviations than most other items. This does not establish deficiencies in these aspects of care, particularly given the generally favourable ratings and pronounced ceiling effects, but it indicates greater heterogeneity in how patients evaluated them. Previous research has emphasised the importance of these aspects of care. Potter et al. reported that poor communication was commonly described in negative physiotherapy evaluations [7]. In patients with lumbar spinal stenosis, Lynch et al. identified a desire for education and individualised care focused on self-management, including clearer explanations of the condition and treatment strategies [26].

Bowling et al. also highlighted the importance of information provision and the fulfilment of expectations in shaping patients’ evaluations of healthcare encounters [27]. The present item-level pattern therefore reinforces the relevance of examining communication and information provision even when overall evaluations are highly favourable.

The observed response pattern may also partly reflect methodological characteristics commonly encountered in patient satisfaction research. Nakhostin Ansari et al. noted pronounced ceiling effects in physiotherapy satisfaction measures [28]. French et al. demonstrated that satisfaction can vary across domains even when composite satisfaction scores remain high [29]. Similarly, Ogwumike et al. highlighted concerns that satisfaction surveys may be insufficiently sensitive to specific care processes, underscoring the value of complementing global or composite measures with more detailed assessment of patients’ experiences [30]. In the present study, the pronounced ceiling effects and skewed distributions, together with greater variability in selected communication- and information-related items, further illustrate the importance of examining item-level responses alongside composite scores.

With respect to the second research question, there was limited evidence that the participant characteristics examined were associated with differences in patient evaluations. Lower educational attainment showed a nominal association with a higher pragmatic composite score in the unadjusted bivariate analysis; however, the effect size was small, and the association did not remain statistically significant after Holm correction for multiple testing. None of the other bivariate comparisons were statistically significant after multiplicity adjustment. Thus, the composite score was relatively homogeneous across the demographic and prior-treatment subgroups examined in this sample.

This limited subgroup differentiation is broadly consistent with Rossettini et al., who emphasised that patient satisfaction reflects interactions among multiple patient, provider, clinical, and contextual factors rather than isolated demographic characteristics [4]. Keramat et al. similarly described patient satisfaction as a complex phenomenon shaped by multiple interacting factors, including patient features, physiotherapist characteristics, and treatment features [10]. In the present study, the selected demographic and prior-treatment predictors explained only a small proportion of variability in the examined outcomes. Factors not included in the present models—such as expectations, treatment outcomes, therapeutic alliance, symptom severity, waiting times, and organisational characteristics—may warrant investigation in future studies.

Some nominal associations emerged in the exploratory item-level analyses. Male sex was nominally associated with higher self-introduction ratings, while previous treatment at the same facility was nominally associated with lower treatment-explanation ratings. The ordinal sensitivity analyses showed the same direction of association for these two findings; however, neither association remained statistically significant after correction for multiple testing. In addition, the nominal linear association for previous treatment at the same facility was not supported by the corresponding bootstrap inference. These item-level findings should therefore be regarded as exploratory signals rather than evidence of established subgroup differences.

Collectively, the findings indicate that relying on a composite score alone may obscure variability across individual questionnaire items. Although the pragmatic composite score was high, communication- and information-related items showed comparatively greater variability. This supports complementing composite measures with item-level assessment when evaluating patients’ experiences of outpatient physiotherapy care. From a quality-improvement perspective, these items may be useful areas for further assessment rather than evidence of specific deficits requiring intervention. Patient-centred communication, explanation of treatment rationale, discussion of potential treatment-related effects, patient education, and shared decision-making remain relevant aspects of physiotherapy practice that may warrant continued attention.

Methodologically, the findings also illustrate challenges inherent in patient satisfaction research. Highly favourable ratings, ceiling effects, and limited response variability can reduce the ability to identify differences between patients and may limit the sensitivity of composite measures. Future research should therefore use validated multidimensional instruments, larger and more diverse samples, and multicentre longitudinal designs to examine the contribution of clinical, interpersonal, organisational, and contextual factors to patient evaluations of outpatient physiotherapy over time.

### Limitations of the Study

Several limitations should be considered when interpreting these findings. First, the cross-sectional design precludes causal inference and captures patient evaluations at a single point during treatment; ratings may also have been influenced by symptoms, expectations, recent treatment experiences, or other transient factors not assessed in this study. Second, this was a single-centre study using convenience sampling, which limits generalisability to other settings and populations. The sample consisted predominantly of middle-aged female participants, limiting subgroup analyses and the precision of some estimates. Although all 155 patients invited to participate returned the questionnaire, the total number of potentially eligible patients attending the department during the recruitment period was not prospectively recorded. Therefore, the extent to which the recruited sample represented the overall eligible patient population could not be determined.

The satisfaction data showed pronounced ceiling effects, limited variability, and skewed distributions, reducing discrimination between participants and the ability to detect small differences. Non-parametric methods, bootstrap inference, and ordinal logistic sensitivity analyses were used where appropriate; however, sparse observations in the lower response categories limited the stability of the ordinal models and precluded reliable assessment of the proportional-odds assumption. Categorisation of selected participant characteristics, particularly age and educational attainment, may also have obscured more granular subgroup differences. The regression models explained only a small proportion of the variability in satisfaction outcomes, and potentially relevant factors such as symptom severity, treatment outcomes, waiting times, therapeutic alliance, expectations, and organisational characteristics were not included. Moreover, none of the individual predictor-level associations remained statistically significant after correction for multiple testing; these findings should therefore be considered exploratory rather than confirmatory.

The study-specific questionnaire had not undergone prior formal psychometric validation. Although exploratory factor analysis and internal consistency analyses were performed, marginal sampling adequacy, pronounced ceiling effects, and sparse lower response categories limited interpretation of its dimensional structure. Consequently, the pragmatic composite process-related satisfaction score should be regarded as a summary indicator rather than as a validated measure of a single underlying construct. The side-effect information item did not include a “not applicable” response option, although its relevance may have varied according to the intervention received; responses to this item should therefore be interpreted cautiously. Self-reported evaluations may also have been influenced by social desirability, gratitude, or reluctance to criticise care. Although questionnaires were anonymous and returned to a designated collection box rather than directly to physiotherapists, these potential sources of response bias cannot be excluded. In addition, individual treating-physiotherapist identifiers were not recorded at the participant level; therefore, potential clustering of responses within physiotherapists could not be examined or accounted for analytically.

Given these limitations and the exploratory nature of the study, the findings should be considered preliminary and hypothesis-generating. Future multicentre and longitudinal studies using validated multidimensional instruments, larger and more diverse samples, and a broader range of clinical, treatment-related, and organisational variables are warranted.

## 5. Conclusions

Patients receiving outpatient physiotherapy in this publicly funded primary care setting reported highly favourable evaluations across process-related aspects of care. Despite the high pragmatic composite score, greater variability was observed for selected communication- and information-related items, particularly self-introduction, side-effect information, and treatment explanation, demonstrating the value of examining item-level responses alongside composite scores. Demographic and prior-treatment characteristics explained only a small proportion of variability in the examined outcomes, and no individual predictor-level association remained statistically significant after correction for multiple testing. These findings suggest that the selected characteristics alone provide limited explanation of differences in patient evaluations and that other clinical, interpersonal, and organisational factors warrant further investigation. Given the exploratory, single-centre design and the methodological characteristics of the satisfaction data, the findings should be considered preliminary and hypothesis-generating. Further multicentre studies using validated multidimensional instruments and including a broader range of potentially relevant factors are needed to better understand patient evaluations of outpatient physiotherapy care.

## Figures and Tables

**Table 1:** Participant characteristics (*N* = 151).

Variable		*n* (%)
Sex	Female	104 (68.9)
	Male	47 (31.1)
Age group	18–47 years	38 (25.2)
	48–67 years	91 (60.3)
	68–87 years	22 (14.6)
Presenting condition	Spine disorder	45 (29.8)
	Mixed multisite musculoskeletal pain	28 (18.5)
	Post-traumatic/postoperative condition	24 (15.9)
	Lower extremity disorder	21 (13.9)
	Upper extremity disorder	19 (12.6)
	Rheumatologic/systemic condition	9 (6.0)
	Neurological condition	3 (2.0)
	Other condition	2 (1.3)
Education level	No formal education	2 (1.3)
	Secondary education	101 (66.9)
	Higher education	19 (12.6)
	University education	29 (19.2)
Employment status	Employed	99 (65.6)
	Retired	45 (29.8)
	Student	2 (1.3)
	Unemployed	5 (3.3)
Previous physiotherapy experience	Yes	43 (28.5)
	No	108 (71.5)
Previous treatment in the same facility	Yes	64 (42.4)
	No	87 (57.6)

Note: Values are presented as frequencies (*n*) and percentages (%).

**Table 2:** Satisfaction items and the pragmatic composite process-related satisfaction score (*N* = 151).

Satisfaction Item	Mean ± SD	Median (IQR)
**Assessment thoroughness**	4.56 ± 0.68	5.00 (1.00)
**Attentiveness**	4.87 ± 0.39	5.00 (0.00)
**Explanation clarity**	4.79 ± 0.49	5.00 (0.00)
**Communication time**	4.57 ± 0.66	5.00 (1.00)
**Patience**	4.81 ± 0.41	5.00 (0.00)
**Self-introduction**	4.38 ± 1.11	5.00 (1.00)
**Assessment explanation**	4.54 ± 0.85	5.00 (1.00)
**Consultation time**	4.62 ± 0.66	5.00 (1.00)
**Active listening**	4.74 ± 0.48	5.00 (0.00)
**Treatment explanation**	4.52 ± 0.86	5.00 (1.00)
**Side-effect information**	4.41 ± 1.02	5.00 (1.00)
**Friendliness**	4.89 ± 0.34	5.00 (0.00)
**Respect for privacy**	4.83 ± 0.44	5.00 (0.00)
**Personalised treatment**	4.84 ± 0.37	5.00 (0.00)
**Professional competence**	4.91 ± 0.29	5.00 (0.00)
**Pragmatic composite process-related satisfaction score**	4.69 ± 0.44	4.87 (0.43)

Note: Values are presented as median, interquartile range (IQR), and mean ± standard deviation (SD). Depending on item wording, questionnaire items were rated either on a five-point evaluative scale (1 = very poor to 5 = excellent) or on a five-point agreement scale (1 = strongly disagree to 5 = strongly agree). Higher scores indicate more favourable evaluations of care.

**Table 3:** Bivariate comparisons of the pragmatic composite process-related satisfaction score according to participant characteristics.

Characteristic	Group, *n*	Mean ± SD	Test Statistic	Effect Size (95% CI)	*p*	pHolm
Sex	Female, 104	4.65 ± 0.46	U = 2120	rrb = 0.133 (−0.066 to 0.321)	0.175	0.525
	Male, 47	4.76 ± 0.38				
Age group	18–47, 38	4.64 ± 0.54	H = 5.390	ε^2^ = 0.036 (0.003–0.136)	0.068	0.272
	48–67, 91	4.74 ± 0.39				
	68–87, 22	4.55 ± 0.43				
Education	Higher, 48	4.59 ± 0.47	H = 5.304	ε^2^ = 0.035 (0.002–0.118)	0.021	0.105
	Lower, 103	4.73 ± 0.42				
Previous physiotherapy	No, 108	4.71 ± 0.39	U = 2324	rrb < 0.001 (−0.203 to 0.201)	0.995	1.000
	Yes, 43	4.63 ± 0.55				
Previous facility	No, 87	4.74 ± 0.34	U = 2927	rrb = −0.051 (−0.234 to 0.135)	0.577	1.000
	Yes, 64	4.61 ± 0.54				

Note: Values are presented as mean ± standard deviation (SD). U = Mann–Whitney U statistic; H = Kruskal–Wallis statistic; rrb = rank-biserial correlation; ε^2^ = rank epsilon-squared. Effect sizes are presented with 95% confidence intervals (CIs). *p*-values are unadjusted; pHolm values were adjusted across the five bivariate comparisons using the Holm method. All bivariate analyses were exploratory.

**Table 4:** Exploratory multiple linear regression analyses of process-related satisfaction outcomes.

Outcome/Predictor		B	SE	95% CI	*p*	pHolm	BCa 95% CI	pboot
Pragmatic composite score	Intercept	4.676	0.104	4.470 to 4.882	<0.001	—	4.444 to 4.887	<0.001
	Male sex	0.091	0.079	−0.065 to 0.247	0.252	1.000	−0.046 to 0.246	0.191
	Age 48–67 years	0.007	0.093	−0.178 to 0.191	0.944	1.000	−0.185 to 0.222	0.936
	Age 68–87 years	−0.194	0.128	−0.447 to 0.059	0.132	1.000	−0.485 to 0.085	0.168
	Lower educational attainment	0.104	0.081	−0.055 to 0.264	0.198	1.000	−0.056 to 0.260	0.194
	Previous physiotherapy experience	−0.001	0.118	−0.235 to 0.232	0.991	1.000	−0.288 to 0.283	0.976
	Previous treatment at the same facility	−0.153	0.110	−0.371 to 0.065	0.168	1.000	−0.417 to 0.067	0.194
Self-introduction	Intercept	4.085	0.256	3.579 to 4.590	<0.001	—	3.406 to 4.670	<0.001
	Male sex	0.488	0.193	0.105 to 0.870	0.013	0.312	0.173 to 0.845	0.001
	Age 48–67 years	0.301	0.228	−0.150 to 0.752	0.190	1.000	−0.152 to 0.876	0.192
	Age 68–87 years	−0.137	0.314	−0.757 to 0.483	0.663	1.000	−0.964 to 0.588	0.721
	Lower educational attainment	0.208	0.198	−0.183 to 0.599	0.294	1.000	−0.196 to 0.637	0.319
	Previous physiotherapy experience	−0.278	0.290	−0.850 to 0.295	0.339	1.000	−0.916 to 0.542	0.485
	Previous treatment at the same facility	−0.197	0.270	−0.730 to 0.337	0.468	1.000	−0.938 to 0.282	0.461
Side-effect information	Intercept	3.970	0.242	3.492 to 4.449	<0.001	—	3.250 to 4.513	<0.001
	Male sex	0.221	0.183	−0.141 to 0.583	0.229	1.000	−0.064 to 0.524	0.135
	Age 48–67 years	0.282	0.216	−0.145 to 0.709	0.194	1.000	−0.174 to 0.882	0.255
	Age 68–87 years	−0.087	0.297	−0.674 to 0.501	0.771	1.000	−0.750 to 0.626	0.807
	Lower educational attainment	0.352	0.187	−0.018 to 0.721	0.062	1.000	−0.061 to 0.805	0.090
	Previous physiotherapy experience	0.092	0.274	−0.449 to 0.634	0.737	1.000	−0.571 to 0.873	0.824
	Previous treatment at the same facility	−0.123	0.256	−0.628 to 0.382	0.632	1.000	−0.817 to 0.397	0.667
Treatment explanation	Intercept	4.304	0.201	3.908 to 4.701	<0.001	—	3.685 to 4.721	<0.001
	Male sex	0.164	0.152	−0.136 to 0.465	0.281	1.000	−0.066 to 0.437	0.181
	Age 48–67 years	0.199	0.179	−0.156 to 0.553	0.270	1.000	−0.155 to 0.637	0.303
	Age 68–87 years	−0.138	0.246	−0.625 to 0.349	0.575	1.000	−0.751 to 0.425	0.631
	Lower educational attainment	0.217	0.155	−0.090 to 0.524	0.164	1.000	−0.071 to 0.581	0.149
	Previous physiotherapy experience	0.362	0.227	−0.087 to 0.812	0.113	1.000	−0.160 to 1.019	0.186
	Previous treatment at the same facility	−0.432	0.212	−0.851 to −0.013	0.043	0.989	−0.988 to 0.025	0.070

Note. *N* = 151. B = unstandardised regression coefficient; SE = standard error; CI = confidence interval; BCa = bias-corrected and accelerated; pboot = bootstrap *p*-value; pHolm = Holm-adjusted *p*-value. Reference categories were female sex, age 18–47 years, higher educational attainment, no previous physiotherapy experience, and no previous treatment at the same facility. BCa 95% CIs and bootstrap *p*-values were obtained from 5000 bootstrap resamples. Holm correction was applied across 24 predictor tests from the four models, excluding intercepts. All analyses were exploratory.

**Table 5:** Proportional-odds ordinal logistic regression sensitivity analyses.

Outcome/Predictor		OR	95% CI	*p*	pHolm
Self-introduction	Male sex	2.74	1.17 to 6.41	0.021	0.372
	Age 48–67 years	0.99	0.38 to 2.55	0.978	1.000
	Age 68–87 years	0.36	0.11 to 1.23	0.103	1.000
	Lower educational attainment	1.50	0.68 to 3.29	0.310	1.000
	Previous physiotherapy experience	0.37	0.11 to 1.24	0.106	1.000
	Previous treatment at the same facility	0.92	0.30 to 2.83	0.878	1.000
Side-effect information	Male sex	1.23	0.57 to 2.64	0.598	1.000
	Age 48–67 years	1.10	0.44 to 2.76	0.843	1.000
	Age 68–87 years	0.51	0.16 to 1.64	0.257	1.000
	Lower educational attainment	1.82	0.85 to 3.87	0.121	1.000
	Previous physiotherapy experience	1.03	0.33 to 3.22	0.963	1.000
	Previous treatment at the same facility	0.95	0.34 to 2.65	0.917	1.000
Treatment explanation	Male sex	1.28	0.58 to 2.82	0.543	1.000
	Age 48–67 years	1.17	0.47 to 2.95	0.732	1.000
	Age 68–87 years	0.45	0.14 to 1.51	0.197	1.000
	Lower educational attainment	1.51	0.70 to 3.26	0.291	1.000
	Previous physiotherapy experience	2.15	0.72 to 6.37	0.169	1.000
	Previous treatment at the same facility	0.35	0.13 to 0.96	0.041	0.704

Note: *N* = 151. OR = odds ratio; CI = confidence interval; pHolm = Holm-adjusted *p*-value. Reference categories were female sex, age 18–47 years, higher educational attainment, no previous physiotherapy experience, and no previous treatment at the same facility. Higher outcome categories represent more favourable evaluations; therefore, OR > 1 indicates higher odds of a more favourable response. Holm correction was applied across 18 predictor tests from the three ordinal logistic regression models. All models were exploratory sensitivity analyses.

## Data Availability

The data that support the findings of this study are available from the corresponding author upon reasonable request. The data are not publicly available due to privacy and ethical restrictions.

## Supplementary Materials

The following supporting information can be downloaded at: https://www.mdpi.com/article/10.3390/healthcare14193310/s1; Table S1: Questionnaire items and response formats used to assess process-related aspects of outpatient physiotherapy care.; Table S2. Item-level response distributions and ceiling effects for the 15-item study-specific questionnaire; Table S3. Exploratory factor analysis and internal consistency of the 15-item study-specific questionnaire.

## Abbreviations

The following abbreviations are used in this manuscript:

OPT	Outpatient physiotherapy
