# Supplementary material for: Patient Satisfaction with Process-Related Aspects of Outpatient Physiotherapy Care: Preliminary Insights from Croatia
Source: Healthcare (Basel). 2026 Oct 4;14(19):3310. doi: 10.3390/healthcare14193310 (PMC13664946; doi:10.3390/healthcare14193310)
Supplement: Supplementary file 1 [file healthcare-14-03310-s001.zip › healthcare-4551022-supplementary.pdf]

healthcare

MDPI

Supplementary Table S1. Questionnaire items and response formats used to assess process-related aspects of outpatient physiotherapy care.

No.	Analytical item label	Questionnaire item	Response format
1	Assessment thoroughness	How would you rate the thoroughness of the questions the physiotherapist asked about your health condition and symptoms?	Evaluative
2	Attentiveness	How would you rate the level of attention the physiotherapist gave you when you spoke to them?	Evaluative
3	Explanation clarity	How would you rate the clarity and detail of the explanations provided by the physiotherapist regarding your health problems or proposed procedures?	Evaluative
4	Communication time	How would you rate the duration of the conversation and the amount of time the physiotherapist devoted to communicating with you?	Evaluative
5	Patience	How would you rate the level of patience demonstrated by the physiotherapist during conversations with you?	Evaluative
6	Self-introduction	The physiotherapist introduced themselves during our first interaction.	Agreement
7	Assessment explanation	At the beginning of treatment, the physiotherapist clearly explained the purpose of the assessment tests and procedures.	Agreement
8	Consultation time	During physiotherapy, I had enough time for discussion and consultation with the physiotherapist.	Agreement
9	Active listening	The physiotherapist listened carefully while I described my health problems.	Agreement
10	Treatment explanation	The physiotherapist explained in detail the results and significance of specific treatment procedures.	Agreement
11	Side-effect information	The physiotherapist informed me about possible side effects that could occur during treatment.	Agreement
12	Friendliness	The physiotherapist was friendly and approachable.	Agreement
13	Respect for privacy	The physiotherapist respected my privacy.	Agreement
14	Personalised treatment	The physiotherapist took my needs and abilities into account.	Agreement
15	Professional competence	The physiotherapist appeared professional and competent.	Agreement

Note: Items 1–5 used a five-point evaluative response format (1 = Very poor; 2 = Poor; 3 = Satisfactory; 4 = Good; 5 = Excellent), whereas Items 6–15 used a five-point agreement response format (1 = Strongly disagree; 2 = Disagree; 3 = Neither agree nor disagree; 4 = Agree; 5 = Strongly agree). Higher scores indicated more favourable evaluations of care. No items were reverse-coded.

Supplementary Table S2. Item-level response distributions and ceiling effects for the 15-item study-specific questionnaire.

Questionnaire item	1, n (%)	2, n (%)	3, n (%)	4, n (%)	5, n (%)	Ceiling, n (%)
Assessment thoroughness	0 (0.0)	3 (2.0)	7 (4.6)	43 (28.5)	98 (64.9)	98 (64.9)
Attentiveness	0 (0.0)	0 (0.0)	3 (2.0)	13 (8.6)	135 (89.4)	135 (89.4)
Explanation clarity	0 (0.0)	1 (0.7)	3 (2.0)	22 (14.6)	125 (82.8)	125 (82.8)
Communication time	1 (0.7)	0 (0.0)	8 (5.3)	45 (29.8)	97 (64.2)	97 (64.2)
Patience	0 (0.0)	0 (0.0)	1 (0.7)	27 (17.9)	123 (81.5)	123 (81.5)
Self-introduction	9 (6.0)	3 (2.0)	11 (7.3)	27 (17.9)	101 (66.9)	101 (66.9)
Assessment explanation	3 (2.0)	3 (2.0)	8 (5.3)	33 (21.9)	104 (68.9)	104 (68.9)
Consultation time	1 (0.7)	2 (1.3)	3 (2.0)	41 (27.2)	104 (68.9)	104 (68.9)
Active listening	0 (0.0)	0 (0.0)	3 (2.0)	33 (21.9)	115 (76.2)	115 (76.2)
Treatment explanation	3 (2.0)	4 (2.6)	6 (4.0)	36 (23.8)	102 (67.5)	102 (67.5)
Side-effect information	7 (4.6)	1 (0.7)	15 (9.9)	28 (18.5)	100 (66.2)	100 (66.2)
Friendliness	0 (0.0)	0 (0.0)	1 (0.7)	15 (9.9)	135 (89.4)	135 (89.4)
Respect for privacy	0 (0.0)	1 (0.7)	1 (0.7)	20 (13.2)	129 (85.4)	129 (85.4)
Personalised treatment	0 (0.0)	0 (0.0)	0 (0.0)	24 (15.9)	127 (84.1)	127 (84.1)
Professional competence	0 (0.0)	0 (0.0)	0 (0.0)	14 (9.3)	137 (90.7)	137 (90.7)

Note. N = 151 for all questionnaire items, with no missing item-level responses. Values are presented as frequency, n (percentage). Items 1–5 (Assessment thoroughness, Attentiveness, Explanation clarity, Communication time, and Patience) used a five-point evaluative response format: 1 = Very poor, 2 = Poor, 3 = Satisfactory, 4 = Good, and 5 = Excellent. Items 6–15 (Self-introduction through Professional competence) used a five-point agreement response format: 1 = Strongly disagree, 2 = Disagree, 3 = Neither agree nor disagree, 4 = Agree, and 5 = Strongly agree. Higher scores therefore consistently represent more favourable evaluations of care across both response formats. No items were reverse-coded. The ceiling column represents the number and percentage of participants selecting the maximum possible response category (score = 5) for each item. Maximum-category responses ranged from 64.2% for Communication time to 90.7% for Professional competence, demonstrating pronounced ceiling effects across the questionnaire. Percentages may not sum to exactly 100% because of rounding.

Supplementary Table S3. Exploratory factor analysis and internal consistency of the 15-item study-specific questionnaire.

Panel A. Exploratory factor analysis: item loadings and uniqueness	Factor 1	Factor 2	Factor 3	Uniqueness
Assessment thoroughness	—	0.660	—	0.262
Attentiveness	—	0.954	—	0.089
Explanation clarity	—	0.826	—	0.097
Communication time	—	0.647	—	0.154
Patience	—	0.701	—	0.137
Self-introduction	0.757	—	—	0.275
Assessment explanation	0.842	—	—	0.193
Consultation time	0.415	—	—	0.324
Active listening	—	—	—	0.236
Treatment explanation	0.824	—	—	0.113
Side-effect information	0.958	—	—	0.132
Friendliness	—	—	0.691	0.247
Respect for privacy	—	—	0.877	0.160
Personalised treatment	—	—	0.633	0.317
Professional competence	—	—	0.456	0.156

Panel B. Factor retention, variance, correlations, sampling adequacy, and model fit	Factor 1	Factor 2	Factor 3	Additional result
Parallel analysis: observed eigenvalue	10.352	0.888	0.436	Factor 4 = 0.203
Parallel analysis: simulated eigenvalue	0.757	0.474	0.371	Factor 4 = 0.291
Retained	Yes	Yes	Yes	Factor 4 = No
Unrotated eigenvalue	10.463	1.062	0.582	—
Proportion of variance	0.698	0.071	0.039	—
Cumulative proportion of variance	0.698	0.768	0.807	—
Rotated sum of squared loadings	4.560	4.494	3.053	—
Rotated proportion of variance	0.304	0.300	0.204	—
Rotated cumulative proportion	0.304	0.604	0.807	—
Factor correlation: Factor 1	1.000	0.711	0.513	—
Factor correlation: Factor 2	0.711	1.000	0.645	—
Factor correlation: Factor 3	0.513	0.645	1.000	—
Overall KMO (MSA)	0.510	—	—	—
Bartlett's test of sphericity	χ^2 = 8838.047	df = 105	$p < 0.001$	—
Model χ^2	6154.239	df = 63	$p < 0.001$	—
RMSEA (90% CI)	0.800 (0.786–0.820)	—	—	—
SRMR	0.036	—	—	—
TLI	−0.179	—	—	—
CFI	0.303	—	—	—
BIC	5838.150	—	—	—

Panel C. Scale-level internal consistency and item-rest correlations	Estimate	SE	95% CI
Cronbach's α, raw	0.914	0.008	0.899–0.930
McDonald's ω, raw	0.927	0.009	0.909–0.944
Cronbach's α, standardised	0.929	0.008	0.913–0.945
McDonald's ω, standardised	0.930	0.009	0.914–0.946
Average inter-item correlation	0.467	—	—

Assessment thoroughness	0.700	—	0.609–0.774
Attentiveness	0.608	—	0.497–0.700
Explanation clarity	0.709	—	0.619–0.780
Communication time	0.740	—	0.658–0.804
Patience	0.627	—	0.519–0.715
Self-introduction	0.697	—	0.605–0.771
Assessment explanation	0.730	—	0.646–0.797
Consultation time	0.688	—	0.593–0.763
Active listening	0.695	—	0.602–0.770
Treatment explanation	0.803	—	0.738–0.853
Side-effect information	0.717	—	0.629–0.786
Friendliness	0.514	—	0.386–0.623
Respect for privacy	0.424	—	0.284–0.547
Personalised treatment	0.464	—	0.329–0.581
Professional competence	0.646	—	0.542–0.730

Note: EFA = exploratory factor analysis; KMO = Kaiser–Meyer–Olkin measure of sampling adequacy; MSA = measure of sampling adequacy; RMSEA = root mean square error of approximation; SRMR = standardised root mean square residual; TLI = Tucker–Lewis index; CFI = comparative fit index; BIC = Bayesian information criterion; SE = standard error; CI = confidence interval. EFA was based on a polychoric correlation matrix. Factor retention was determined using factor-based parallel analysis, and oblimin rotation was applied to allow correlations among factors. Factor loadings not displayed in the analysis output are represented by an em dash (—). Three factors were retained because the first three observed eigenvalues exceeded the corresponding simulated eigenvalues, whereas the fourth observed eigenvalue did not. Overall sampling adequacy was marginal (KMO = 0.510). Given the marginal sampling adequacy, pronounced ceiling effects, sparse lower response categories, and unfavourable global fit indices, the factor solution was considered exploratory and was not used to define or score separate subscales. Raw and standardised internal consistency coefficients are reported separately. Cronbach's α, McDonald's ω, and the average inter-item correlation were interpreted as indices of internal consistency and not as evidence of unidimensionality or construct validity.
